# Nonradiative Relaxation Mechanisms of UV Excited Phenylalanine Residues: A Comparative Computational Study

**DOI:** 10.3390/molecules22030493

**Published:** 2017-03-21

**Authors:** Momir Mališ, Nađa Došlić

**Affiliations:** 1 Ruđer Bošković Institute, HR-10000 Zagreb, Croatia; 2 Ecole polytechnique fédérale de Lausanne, CH-1015 Lausanne, Switzerland

**Keywords:** nonadiabatic molecular dynamics, nonradiative deactivation, peptide, phenylalanine

## Abstract

The present work is directed toward understanding the mechanisms of excited state deactivation in three neutral model peptides containing the phenylalanine residue. The excited state dynamics of the $\gamma_{L}\left(g+\right)$ folded form of *N*-acetylphenylalaninylamide (NAPA B) and its amide-*N*-methylated derivative (NAPMA B) is reviewed and compared to the dynamics of the monohydrated structure of NAPA (NAPAH). The goal is to unravel how the environment, and in particular solvation, impacts the photodynamics of peptides. The systems are investigated using reaction path calculations and surface hopping nonadiabatic dynamics based on the coupled cluster doubles (CC2) method and time-dependent density functional theory. The work emphasizes the role that excitation transfer from the phenyl $\pi \pi^{*}$ to amide $n\pi^{*}$ state plays in the deactivation of the three systems and shows how the ease of out-of-plane distortions of the amide group determines the rate of population transfer between the two electronic states. The subsequent dynamics on the $n\pi^{*}$ state is barrierless along several pathways and leads to fast deactivation to the ground electronic state.

## 1. Introduction

Proteins absorb ultraviolet (UV) light. In the near-UV spectral range (250–280 nm) the absorption arises from the side chains of aromatic amino acids: tryptophan (Trp), tyrosine (Tyr) and phenylalanine (Phe). At shorter wavelengths, the absorption originates from the terminal amino and carboxyl groups, as well as from the amides making up the peptide backbone [1]. The lowest excited states of the amide groups correspond to $n\pi^{*}$ states with excitation localized on the carbonyl [2,3,4]. Formally, the nonbonding electron pair (*n*) belongs to the oxygen atom, and the $\pi^{*}$ is the anti-bonding C=O orbital. These weak transitions are followed by intense $\pi \pi^{*}$ transitions of the C=O groups in the far-UV. The lowest valence transitions of proteins are only slightly shifted compared to the absorption of the bare chromophores, indole (Trp), phenol (Tyr) and phenyl (Phe), but their fluorescence properties are very different from those of isolated chromophores [1]. This indicates that efficient pathways of nonradiative deactivation (NRD) arise from the interaction of the absorbing chromophore and the peptide backbone.

This article deals with the processes that takes place upon light absorption in three small peptide systems containing the Phe residue. Compared to the photophysics of Trp, which has been much studied in the context of protein structure and dynamics [5,6,7,8], the photophysics of Phe has attracted less attention due to the almost negligible fluorescence yield. However, taking into account that Phe is more frequent in the proteome than Trp (three-times so in vertebra), the NRD mechanisms that set up after photoexcitation of the Phe residue may be important for the stability and functionality of proteins.

**Scheme 1:**
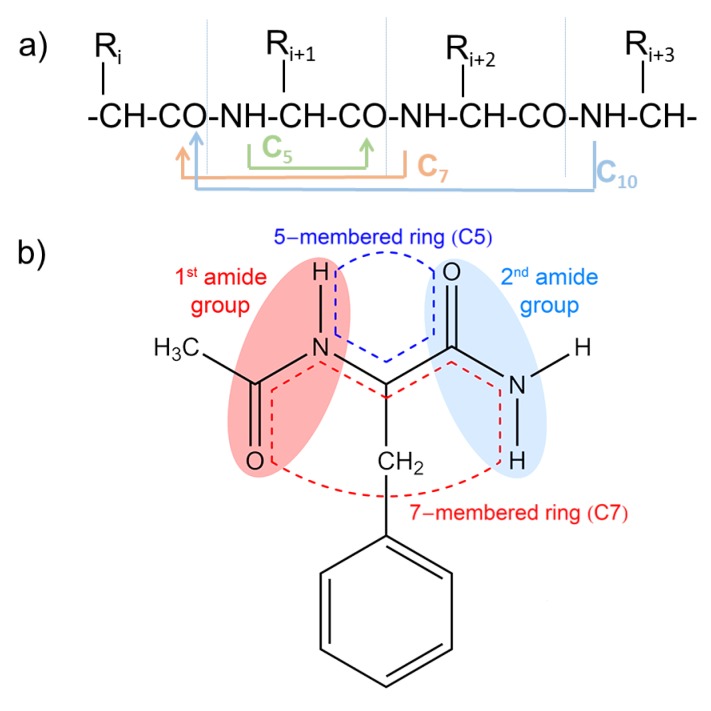
(**a**) H-bonding interactions defining the secondary structures of a peptide chain; (**b**) Structural formula of *N*-acetylphenylalanylamide (NAPA). Interactions characteristic for $\beta_{L}$ (blue dashed line) and *γ* (red dashed line) secondary structures.

Focusing on the theoretical achievements in the field, it was found that the Franck–Condon geometry of formamide is located on a steep part of the $n\pi^{*}$ potential energy surface (PES) and that relaxation toward the minimum of the S_1_ state leads to significant C=O bond extension and deplanarization of the initial structure [9,10]. Complete active space self-consistent field (CASSCF) nonadiabatic (NA) dynamics simulations, however, revealed that the dominant NRD channel does not involve C=O bond dissociation, but fission of the central C–N bond, leading to the formation of CHO and NH_2_ radicals [10]. An important step toward understanding NRD mechanisms in peptides was the paper on the deactivation of excited caped Gly trimer in a *β*-turn conformation by Sobolewski and Domcke [11]. In a *β*-turn, a H-bond connects the *i*-th residue with the (*i* + 3)-th residue forming a 10-member ring and stabilizing the peptide conformation (see Scheme 1).

Using the second-order approximate coupled cluster singles and doubles (CC2) method [12,13], the authors have shown that starting from the $n\pi^{*}$ state with excitation localized on the last amide group of the sequence, H-transfer from the nitrogen to oxygen site leads to a conical intersection (CI) with a charge transfer state that is repulsive along the N–H stretching coordinate [11]. Motion along this state is strongly destabilizing for the ground electronic state, and the CI between the ground and first excited state is easily reached. Once in the ground electronic state, the H atom is transferred back to the nitrogen, essentially converting electromagnetic radiation into molecular vibrations. While the role of amide excited states in the deactivation was clearly demonstrated, it remained unclear how the low-lying excited states localized on the chromophoric side chains of aromatic amino acids couple to the amide excited states. In a subsequent paper, the same deactivation scheme was used to explain the absence of the resonant two-photon ionization (R2PI) signal from a set of Gly-Phe-Ala tripeptide secondary structures predicted to co-exist in the gas phase [14]. Namely, Valdes et al. argued that the absence of these conformations in the R2PI spectrum was due to a sub-picosecond NRD mechanism, which depopulated the Phe $\pi \pi^{*}$ state before the second laser pulse could photoionize it [15]. The same argument was used to explain the absence of certain conformers of Trp-Gly and Trp-Gly-Gly peptides [16]. In the critical conformers, the peptide backbone formed a *γ*-turn. Although the *γ*-turn secondary structure is quite different from the *β*-turn, in both structures, two interfacing amide groups are joined by a H-bond. Therefore, the electron-driven proton transfer mechanism was expected to play an active role in the deactivation of Gly-Phe-Ala. In Gly-Phe-Ala, the initially excited state (S_1_) is the phenyl $\pi \pi^{*}$ state. However, two types of minima have been found on the S_1_ surface, a $\pi \pi^{*}$ benzene type minimum and a $n\pi^{*}$ minimum localized on the first amide group (local excitation (LE) state in the terminology of the authors). Extension of the N–H distance leads to CI with a charge transfer (CT) state with excitations from the second to the first amide group. Unlike the $\pi \pi^{*}$ and LE exited states, the CT state is repulsive along the N–H stretching coordinate. Once in the CT state, a further increase of the N–H distance leads to the CI with the ground electronic state. Again, once in the ground state, the H atom is transferred back to the N atom, and the excess of energy is redistributed as heat.

**Figure 1:**
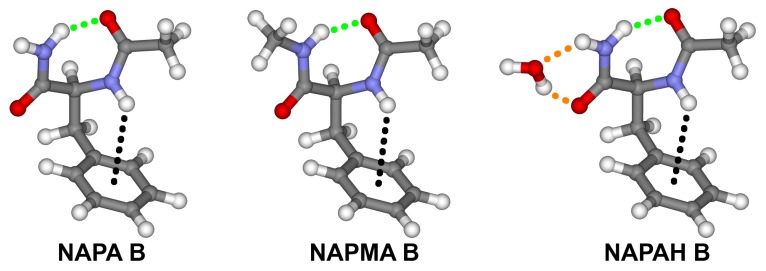
Chemical structure of the three systems under investigation: *N*-acetylphenylalanylamide (NAPA, **left**); *N*-acetylphenylalanylmethylamide (NAPMA, **middle**) and *N*-acetylphenylalanylamide monohydrate (NAPAH, **right**) conformers B ($\gamma_{L}\left(g+\right)$). Dots denote hydrogen bonding interactions.

More recently, we have studied the photophysics of several conformers of two capped dipeptides containing the Phe residue (see Figure 1). *N*-acetylphenylalanineamide (NAPA) and its amide-*N*-methylated derivative *N*-acetylphenylalanylmethylamide (NAPMA) display a range of deactivation mechanisms [17,18] also at play in larger peptides [19]. This paper reviews these mechanisms and sets the ground for the study of NRD mechanisms in NAPA monohydrates (see Figure 1). The goal of the paper is to unravel how the environment in general, and solvation in particular, impacts the intrinsic deactivation pathways of Phe-containing peptides.

## 2. General Aspects

**Figure 2:**
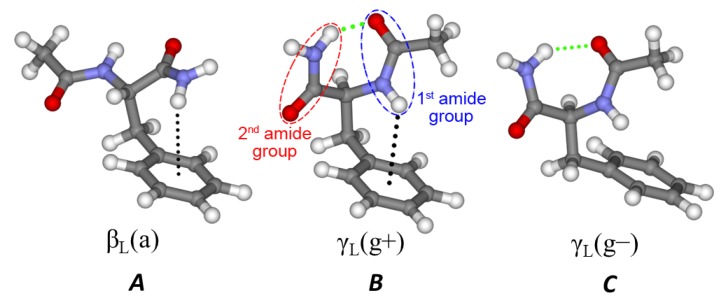
NAPA conformers **A** ($\beta_{L}\left(a\right)$); **B** ($\gamma_{L}\left(g+\right)$) and **C** ($\gamma_{L}\left(g-\right)$). The green dotted lines represent the N–H···O H-bonds within the seven-member $\gamma_{L}$ rings of NAPA conformers B and C, while the black dotted line indicates the N_*Phe*_–H···π interaction in NAPA conformer B.

Conformer-specific measurements are essential to unequivocally connect the photophysical behavior of a peptide and its structure. For an overview of experimental achievements, the reader is advised to consult some of excellent experimental reviews [20,21,22,23]. The description of the experimental setups and relevant measurements performed on the systems under investigation can be found in [17,24,25]. Basically, the molecules are transferred to the gas phase by laser desorption from a solid sample and cooled in a supersonically-expanding jet of argon gas to an estimated temperature of a few Kelvins (<10 K). Under such conditions, the molecules can be cooled down by inelastic collisions with carrier gas atoms to their most stable configurations with very little excess of internal energy. Molecules and complexes are then analyzed by IR/UV double-resonance spectroscopy. Assignment of the observed species is supported by electronic structure computations [26,27,28,29]. For NAPA, only three conformers were detected. They were identified by comparing their N–H (amide A) and C=O (amide I) stretching frequencies with ones calculated for a set of theoretically-predicted NAPA conformers [26,27]. These conformers, denoted A, B and C, are shown in Figure 2. Conformer A belongs to a class of $\beta_{L}$ strands characterized by Ramachandran angles Ψ and Φ of 180°, whereas conformers B and C belong to a class of equatorial $\gamma_{L}$-turns secondary structures ($\Psi =+50^{\circ }$, $\Phi =-75^{\circ }$). The main structural difference between the latter two is in the relative position of the phenyl ring with respect to the backbone, which are accordingly designated as *gauche*(+) and *gauche*(−) structures depending on the relative orientation to the N–C_*α*_ bond in the first amide group. In this respect, the phenyl ring of conformer A phenyl is oriented *anti*. The three experimentally-detected conformers of NAPMA are analogous to those of NAPA.

From Figure 2, it is evident that the phenyl ring experiences different local environments in conformers A, B and C. These different environments are not only responsible for the shift in the position of absorption lines, but may also be responsible for the strikingly different excited state lifetimes of the three species. Table 1 collects the excited state lifetimes of selected isotopomers of the three conformers of NAPA and NAPMA as obtained from nanosecond pump-probe experiments [17,18]. Measurements, performed at the origin of the $\pi \pi^{*}$ transition, revealed that the lifetime of NAPA conformer B is much shorter (50-times) than that of the other two conformers. Further, it was found that deuteration has an almost negligible effect on the lifetimes, but that a remarkable prolongation of the lifetime of conformer B is obtained by methylation of the second amide group [18]. These striking differences will be addressed in the following sections.

The $\beta_{L}\left(a\right)$, $\gamma_{L}\left(+\right)$ and $\gamma_{L}\left(-\right)$ conformers observed in NAPA and NAPMA are also found in the four experimentally-observed NAPA monohydrates [25]. Basically, monohydrates can be regarded as particular NAPA conformers with a water molecule bonded to a specific functional group. To facilitate the comparison with NAPA and NAPMA, we will refer to the NAPA monohydrate Y conformer of Biswal et al. as NAPAH B since the peptide part of the NAPA Y is structurally identical to the NAPA B conformer [25].

## 3. Results and Discussion

### 3.1. Electronic Structure

We first analyze the geometrical differences between NAPA, NAPMA and NAPAH conformers B. If we consider the NH_2_–C(=O)– group of NAPA conformer B as the unperturbed system, the perturbation is caused by the substitution of the distal hydrogen atom (H_*dist*_) by the methyl group in NAPMA, or in the case of NAPAH by binding of a water molecule. The perturbation is then transferred to the rest of the molecules mostly by intramolecular dispersive interactions.

**Figure 3:**
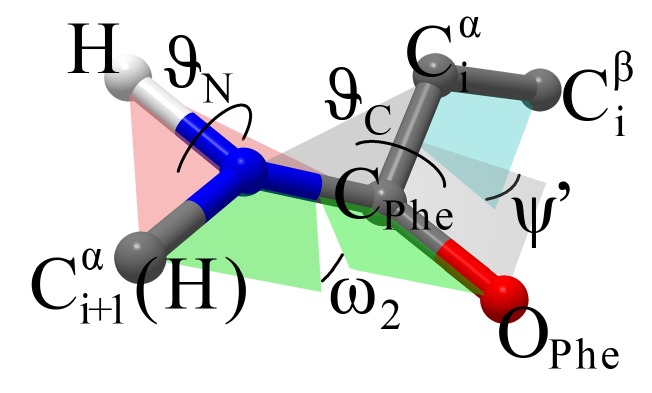
Possible distortions of the second amide group and the definition of the torsional angles $\vartheta_{C}$, $\vartheta_{N}$, $\omega_{2}$ and $\psi^{\prime }$.

The water molecule perturbs the second amide group more than the covalently-attached methyl group in NAPMA. The two hydrogen bonds, $O_{H_{2}O}$–H···O_(*II*)_ and $O_{H_{2}O}\cdot \cdot \cdot$H_*dist*_–N_(*II*)_, cause a 0.014 Å extension of the C=O bond and a 0.013 Å compression of the C–N with respect to the NAPA values. In NAPMA B, these bond distances change by only 0.005 and −0.001 Å respectively. Furthermore, in NAPA B, the second amide NH_2_ group is pyramidalized by an angle of $\vartheta_{N}=tor\left(H_{dist}-N_{\left(II\right)}-H-C_{\left(II\right)}\right)$ of −159.4 degrees (see Figure 3). In both NAPMA and NAPAH, the NH_2_ group is planar.

Although the differences in the geometry of the second amide group upon methylation or hydration are significant, the effect on the Ac–Phe frame is contained. Root mean square deviations (RMSD) between NAPMA/NAPA and NAPAH/NAPA Ac–Phe frames are only ∼0.05 Å.

The excitation energies are consistent with the geometrical differences between NAPA, NAPMA and NAPAH. Table 2 compares vertical absorption energies, as well as adiabatic and zero point energy (ZPE)-corrected absorption energies of the four lowest singlet states of the conformer B of NAPA, NAPMA and NAPAH. Results were obtained at the CC2 and multi-state complete-active-space second-order perturbation theory (MS-CASPT2) levels of theory. See also reference [27] for TDDFT and MS-CASPT2 absorption energies and oscillator strengths in the four most stable NAPA conformers.

**Figure 4:**
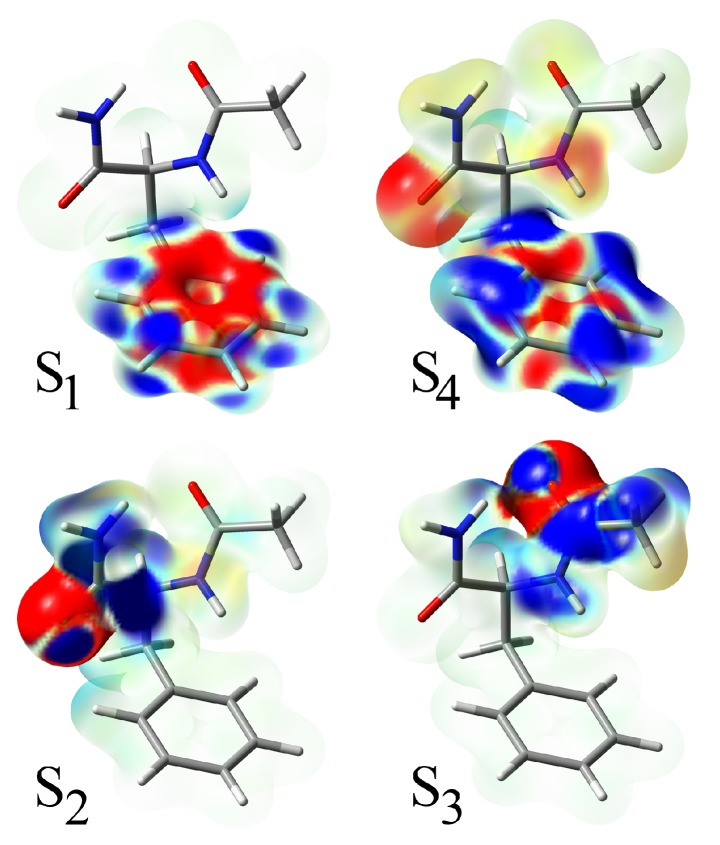
Charge migration in NAPA conformer B upon vertical excitation. CC2 electron density differences between the excited and ground electronic states mapped on the ground state total density isosurface of 0.02 Å^−3^. The red (blue) areas indicate depletion (increase) of electron density in the excited state with respect to the ground state. Upper panel S_1_ $\left(\pi \pi^{*}\right)$ and S_4_ $\left(\pi \pi^{*}\right)$; lower panel S_2_ $\left(n\pi_{\left(II\right)}^{*}\right)$ and S_3_ $\left(n\pi_{\left(I\right)}^{*}\right)$.

In all species, the four lowest states include two $\pi \pi^{*}$ states and two $n\pi^{*}$ states. The electron density differences between the excited and the ground state are shown in Figure 4. The lowest excitation of the $\pi \pi^{*}$ type is localized on the phenyl ring and is analogous to the $B_{2u}$ state of benzene. Comparing the excitation in NAPA, NAPMA and NAPAH, it is evident that the state is virtually unaffected by the chemical motif of the second amide group. The same is true for the second $\pi \pi^{*}$ transition (S_4_), the one analogous to the $B_{1u}$ state of benzene. Density differences reveal that at the CC2 level, this states contains about a 14% contribution of the next S_5_ state, which is a charge transfer state located just 0.2 eV above S_4_.

Intercalated between the two $\pi \pi^{*}$ states are two $n\pi^{*}$ states, $n\pi_{\left(I\right)}^{*}$ and $n\pi_{\left(II\right)}^{*}$ with excitations localized on the first and second amide group, respectively. In NAPA and NAPMA, the second excited state is $n\pi_{\left(II\right)}^{*}$. In NAPAH, however, binding of water significantly affects the second amide group and destabilizes the $n\pi_{\left(II\right)}^{*}$ excited state, which exchanges order with the $n\pi_{\left(I\right)}^{*}$ state, both at the CASPT2 and RI-CC2 levels. The weaker perturbation in NAPMA B is not sufficient to cause the same effect.

Adiabatic excitations are more suitable for comparison with experiments. Geometry optimization of the S_1_ $\left(\pi \pi^{*}\right)$ state at the CC2 level leads to a minimum (further designated as $M_{\pi \pi^{*}}$) that slightly differs from the ground state one. In all three $M_{\pi \pi^{*}}$ minima, the C–C bonds of the phenyl ring are longer on average by 0.035 Å with respect to their ground state values. The elongation of the phenyl C–C bonds is slightly smaller than 0.040 Å as obtained in benzene by reference CASPT2 calculations, even though the absolute values of phenyl C–C and C–H bond lengths of 0.011 and 0.014 Å, respectively, are longer than the corresponding benzene values [30]. The expansion of the phenyl ring accounts for the 0.2 eV energy difference between the vertical and adiabatic excitation energies. The ordering of the other excited states remained unaltered by the geometry optimization of the S_1_ state.

The electronic properties of the first $\pi \pi^{*}$ state are completely analogous to the toluene B_2_ state, whose experimental 4.65 eV ^1^A_1_ → ^1^B_2*u*_ vibrationless ($0_{0}^{0}$) transition is almost isoenergetic to the measured transitions of the NAPA systems [31]. To reproduce the experimental values, nuclear ZPE contributions were included to simulate the vibronic states. Because of the expansion of the phenyl ring in the $M_{\pi \pi^{*}}$ minimum, most harmonic modes are red-shifted with respect to their ground state values. This stabilizes the $\pi \pi^{*}$ vibronic state by ∼0.2 eV and brings the calculated values at the RI-CC2/cc-pVDZ level just 0.1 eV above the measured ones. Furthermore, ${6b}_{0}^{1}$ vibronic excitations were measured 530 cm^−1^ above the UV origin in both NAPA and NAPMA conformers B [17,19]. As the corresponding vibronic transition in toluene is found at 532 cm^−1^ [31], we assigned the transitions to the 528 and 521 cm^−1^ in-plane distortion normal modes, of NAPA and NAPMA, respectively.

The CASPT2 method yields exactly the same states in all ground and S_1_ minima. The inversion of the $n\pi_{\left(II\right)}^{*}$ and $n\pi_{\left(I\right)}^{*}$ states in NAPAH B is also reproduced. Compared to corresponding RI-CC2 value, the first excitation is significantly red-shifted, while the fourth excited state is strongly blue-shifted. No state of the CT character was observed among the higher roots at the multireference level, but CC2 calculations suggest that it would be necessary to include the free electron pairs of the Phe nitrogen atom in the active space to adequately describe the CT character.

Focusing on the two $n\pi^{*}$ states, population analyses and electron density differences confirm that electron redistribution takes place from the oxygen to the carbon atom of the C=O group. A small increase of the electronic density on the neighboring nitrogen atom is also observed, in contrast to the reported depletion of the electronic charge on the N atom in simple amides by Serrano-Andrés et al. [2]. Mulliken population analyses at the CASPT2 level confirm the CC2 results and indicate breaking of conjugation between N and C amide atoms upon $n\pi^{*}$ excitation. Both $n\pi_{\left(II\right)}^{*}$ and $n\pi_{\left(I\right)}^{*}$ states posses a number of local minima in which the corresponding C=O bonds are significantly elongated and the whole amide group deplanarized due to the absence of conjugation between the carbon and the nitrogen atom. These minima, found lower in energy than $M_{\pi \pi^{*}}$, play a central role in the excited state deactivation of the three peptides and will be addressed in the following sections.

### 3.2. Investigation of Nonradiative Deactivation Pathways of NAPA B

Before analyzing some of the relaxation pathways that emerged from nonadiabatic dynamics simulations, it is useful to comment on the performance of the TD-PBE0/cc-pVDZ method. The TD-PBE0/cc-pVDZ vertical excitation energy to the two lowest excited states of 5.393 eV and 5.487 eV (ground state optimized at MP2/cc-pVDZ) compares relatively well with the RI-CC2 values of 5.176 and 5.606 eV in NAPA conformer B. However, TD-PBE0 groups the higher excited states more densely than RI-CC2. In particular, the energy of the second $\pi \pi^{*}$ state, which has a significant charge transfer contribution arising from the mixing with the S_5_ state, is significantly underestimated and falls below the $n\pi^{*}$ state. The problem becomes severe upon optimization of S_1_ states. The $\pi \pi^{*}$ PES is dissociative along the N_*Phe*_–H bond stretching coordinate, and the hydrogen atom from the N_*Phe*_–H group is transferred to the *ortho* C atom of the phenyl ring. This causes a rise in the ground state energy, and a S_1_/S_0_ CI is reached.

**Scheme 2:**
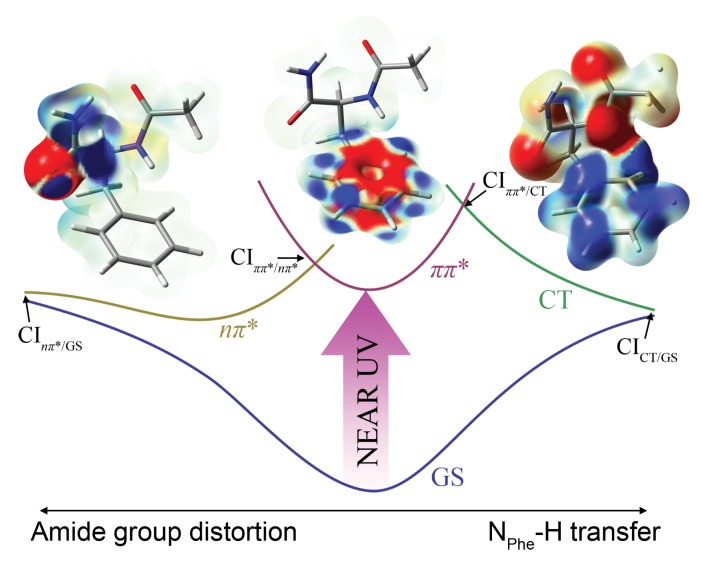
Illustration of the three decay mechanism of NAPA B obtained from nonadiabatic dynamics simulations. Mechanism I involves H-transfer transfer to the closest C atom of the phenyl ring, whereas Mechanisms II and III involve deformation of the second and first amide group, respectively. The arrow indicates the excitation to the minimum of the $\pi \pi^{*}$ state. The insets show the electron density differences between the electronic excited states relevant to the three mechanisms and the ground state. The red (blue) areas indicate depletion (increase) of electron density in the excited state with respect to the ground state.

In the dynamics simulations, we propagated a set of 44 NA-TDDFT trajectories starting from the first excited state assuming a vertical excitation of ground state geometries. Depending on the initial geometry, the S_1_ state corresponds either to the $\pi \pi^{*}$ or the $n\pi^{*}$ state. Taking into account the limitations of TDDFT, nonadiabatic dynamics simulations have an exploratory character, and the deactivation mechanisms indicated by the simulations need to be refined at the more adequate CC2 level. Mechanism I involves H-transfer to the closest C atom of the phenyl ring. While there is a growing body of evidence indicating that H-transfer to carbon atoms of aromatic rings may be a very efficient photochemical reaction [32,33], in NAPA $\gamma_{L}\left(g+\right)$, this pathway is likely to be overrepresented in the simulation (11 out of 44 trajectories) owing to the shortcomings of TD-PBE0. Mechanism II involves as a crucial step a CI between the initially excited $\pi \pi^{*}$ state and the $n\pi_{\left(II\right)}^{*}$ with excitation localized on the second amide group. This is the dominant deactivation mechanism according to nonadiabatic dynamics simulations encountered in 25 out of 44 trajectories. Mechanism III, encountered in four trajectories, operates similarly to Mechanism II, but involves internal conversion from the $\pi \pi^{*}$ state to the $n\pi_{\left(I\right)}^{*}$ with excitation localized on the first amide group (N-terminus). Once in the $n\pi_{\left(I\right)}^{*}$ state, deactivation to the ground state proceeds either by H-transfer along the N_*Phe*_–H⋯O=C_*Ac*_ bond or by elongation of the C_*Ac*_=O bond. The former is actually the realization of the electron driven H-transfer mechanism of Domcke and Sobolewski in NAPA [14]. In both Mechanisms II and III, after leaving the initially excited $\pi \pi^{*}$ state, the system requires no additional energy to return to the ground state. The three deactivation mechanisms are illustrated in Scheme 2. The electron density differences characteristic for Mechanism I and Mechanism II are shown in the insets.

**Figure 5:**
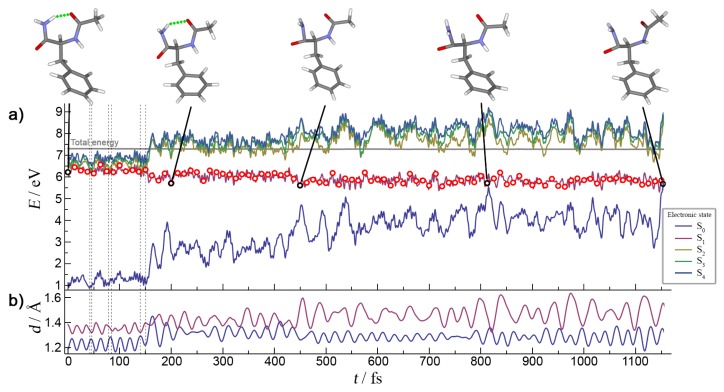
(**a**) Time evolution of the ground and four lowest excited adiabatic electronic states along the trajectory exhibiting Mechanism II. The red circles designate the currently-occupied state. Insets show selected geometries along the trajectory (black circle). Vertical dashed lines indicate the occurrence of trajectory surface hops. The energies are relative to the NAPA conformer B minimum energy; (**b**) The change of C_*Phe*_=O (blue) and C_*Phe*_–N (bordeaux) bond lengths along the trajectory.

**Figure 6:**
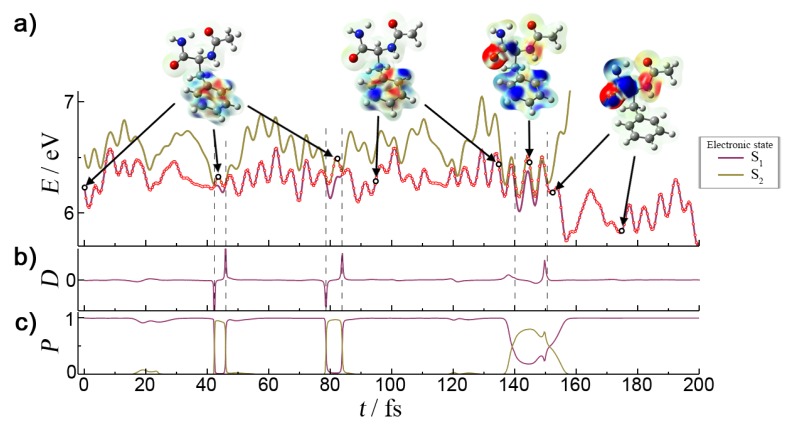
Details of the first 200 fs of the trajectory displayed in Figure 5. (**a**) Close up of S_1_ and S_2_ adiabatic states. The red circles designate the currently classically-occupied state, while the electronic density difference insets designate the state character for a selected trajectory point. Vertical dashed lines indicate the occurrence of trajectory surface hops. The energies are relative to the NAPA conformer B minimum energy. Insets show the charge redistribution in the indicated excited state relative to its ground state. See the caption of Figure 4 for details; (**b**) Dynamical coupling between S_1_ and S_2_ adiabatic states scaled to the absolute maximum value; (**c**) Evolution of S_1_ and S_2_ electronic populations.

Let us discuss Mechanism II in more detail. A characteristic NA trajectory deactivating by Mechanism II is shown in Figure 5. The trajectory starts from a well-defined $\pi \pi^{*}$ state. The population transfer to the $n\pi_{\left(II\right)}^{*}$ state occurs after 150 fs. Figure 6 shows the details of the first 200 fs of the dynamics. In this time span, surface hopping between the neighboring S_1_ and S_2_ states had occurred six times paired in three separate events of the S_1_ → S_2_ → S_1_ type. In the first two, the change of states is so rapid that the electronic populations just swap between the two adiabatic states. This can be seen from the instantaneous increase of dynamical coupling between the states. In these two regions, the electronic population and the characters of the states behave diabatically, and after the second interaction region (t≥ 85 fs), the system still remains in the initial $\pi \pi^{*}$ state. However, the third encounter exhibits a steadily monotonic increase of the dynamical coupling as a consequence of a smooth change of the character of the electronic states. As the electronic density difference shows, the initial $\pi \pi^{*}$ state acquires a $n\pi_{\left(II\right)}^{*}$ type excitation due to mixing between the two states. This gradual mixing arises from the trajectory passing through the S_1_/S_2_ CI, but in a region of the configuration space where the nonadiabatic coupling is smaller than in the previous four cases. Due to the gradual change of dynamical couplings in this region, the S_1_ electronic population displays a monotonic decrease, and the whole transition between states is adiabatic. At 145 fs, the characters of the S_1_ and S_2_ states are almost indistinguishable due to equal mixing of adiabatic electronic states. This equal contribution changes in the following few steps, and the last S_2_ → S_1_ surface hop is more diabatic, as the narrower dynamical coupling shows. This diabaticity of surface hop is necessary to preserve the character of the new state. After 150 fs, the S_1_ state has completely evolved into $n\pi_{\left(II\right)}^{*}$. The energy of the other excited states rises abruptly after 150 fs (see Figure 5a) and soon exceeds the total energy of the system. Thus, they do not influence the dynamics in any way. Once in the $n\pi_{\left(II\right)}^{*}$ state, the benzene ring C–C bonds contract by 0.02 Å, while the C_*Phe*_=O and C_*Phe*_–N bonds expand by 0.18 and 0.12 Å, respectively (Figure 5b). At this point, the H-bond is still preserved. The modest gain in kinetic energy of 0.25 eV is initially channeled into the C_*Phe*_=O and C_*Phe*_–N stretching modes and then redistributed into distortion modes. The initial planarity of the second amide group is lost due to breaking of the conjugation between the C_*Phe*_ and N atoms. Local functional groups involving the two atoms deplanarize, and the O atom moves away from the phenyl ring. Although these geometrical changes only weakly affect the energy of the S_1_ state, the energy of the ground state exhibits a sudden rise of almost 2 eV. At around 300 fs, the NH_2_ twisting and waging motions become so excited that they eventually break the $\gamma_{L}$-ring H-bond. The NH_2_ group can now almost freely rotate around the C_*Phe*_–N bond. After the next 100 fs, part of the C_*Phe*_=O vibrational energy is transferred to the C_*Phe*_–N stretch vibration. The combination of backbone strain and heavy distortion of the second amide group contributes to the ground state instability, due to which the S_0_ state shows large oscillations, and on a few occasions, the S_1_-S_0_ energy gap decreases to less than 0.5 eV. This is also the situation in the last point of the dynamics in which the excited state calculation is aborted due to numerical instabilities in the solution of Cassida’s equation near a true intersection point [34].

### 3.3. Comparative Analysis of the $n\pi_{\left(II\right)}^{*}$ States of NAPA B and NAPMA B

Nonadiabatic molecular dynamics simulations of NAPA B have revealed three deactivation mechanisms starting from the $\pi \pi^{*}$ state. We made no attempts to compute the excited state lifetimes associated with the three mechanisms. The experimental excitation at the origin of the $\pi \pi^{*}$ transition results in an excited state lifetime of 1.5 ns in the case of NAPA B and thus makes such computation unfeasible, even at the TDDFT level. Thus, simulations could not indicate the mechanism responsible for the remarkably shorter lifetime of NAPA B. The absence of any deuteration effect on the experimental lifetimes and the known underestimation of the energy of charge transfer states in TD-PBE0 led us to exclude Mechanism I. To discriminate between Mechanisms II and III, we adopted a different strategy. We supposed that if Mechanism II was the one responsible for the short lifetime of NAPA B, then a chemical modification of the second amide group would lead to changes in the excited state lifetime. Accordingly, we focused on NAPMA B.

In Table 1, we reported the lifetimes of the three NAPMA conformers measured at the origin of the S_1_ $\left(\pi \pi^{*}\right)$ state. For NAPA conformers A, B and C, pump-probe experiments with nanosecond (picosecond) resolution provided lifetimes of 70 ns, <7 ns (1.5 ns) and 35 ns, respectively. Comparing these values to NAPMA A, B and C lifetimes of 67, 48 and 62 ns, it becomes clear that the mechanism responsible for the very short lifetime of NAPA B is likely to be Mechanism II. As the difference in the lifetimes of NAPA B and NAPMA B is striking, it is worth investigating how methylation of the second amide group actually hampers the deactivation of NAPMA via Mechanism II.

Mechanism II involves a transfer of excitation from the $\pi \pi^{*}$ state to the $n\pi_{\left(II\right)}^{*}$ state followed by a set of geometry deformations of the second amide group, which lead to the CI with the ground state. The second part is comparable to acetamide deactivation from the $n\pi^{*}$ state for which a variety of deactivation mechanism have been found [35]. In NAPA and NAPMA, however, the gain in kinetic energy for reaching the $n\pi_{\left(II\right)}^{*}$ state is approximatively half the one in acetamide, implying that only deactivation channels with a low activation barrier contribute to the deactivation of the $n\pi_{\left(II\right)}^{*}$ state. RI-CC2 reaction path calculations indicate that in both NAPA B and NAPMA B, the CI with the ground state is most easily reached by elongation of the C_*Phe*_=O bond. In both systems, this path is barrierless.

Thus, the difference between NAPA B and NAPMA B originates from the first part of Mechanism II governing the population transfer from the $\pi \pi^{*}$ to the $n\pi_{\left(II\right)}^{*}$ state via the $\pi \pi^{*}$/$n\pi_{\left(II\right)}^{*}$ CI seam. To map the seam, we start by searching for local minima on the $n\pi_{\left(II\right)}^{*}$ PESs of NAPA B and NAPMA B. By scanning the three torsional, angles $\vartheta_{C}$, $\vartheta_{N}$ and $\omega_{2}$, defining the second amide group (see Figure 3), we obtained six minima with preserved $\gamma_{L}$ secondary structure. These were grouped into two categories based on the pyramidalization of the C_*Phe*_=O group defined by the $\vartheta_{C}$ angle. The $\vartheta_{C}$ angle basically determines the direction in which the C_*Phe*_=O bond moves from the plane defined by the initial $M_{\pi \pi^{*}}$ structure. Positive (negative) values indicate that the O_*Phe*_ atom moves toward (away from) the phenyl ring. Structures with negative and positive $\vartheta_{C}$ angle values are designated as M_*a*_ and M_*b*_, respectively, while numerical notations 1, 2 and 3 are used for structures within the set. Table 3 compiles the relative energies and most important geometrical parameters of the minima.

Six almost isoenergetic M_(*II*)_ minima were found 0.62–0.76 eV lower in energy than the reference $M_{\pi \pi^{*}}$ minimum. This large stabilization stems from a major distortion of the second amide group, which encompasses torsion ($\omega_{2}$) and pyramidalization of the amide N and carbonyl C atoms, as well as by a 0.14–0.23 Å elongation of the C=O bond. Apart from the distortion of the second amide group, the rest of the molecular framework deviates only slightly from $M_{\pi \pi^{*}}$, implying that the seam of $\pi \pi^{*}$/$n\pi_{\left(II\right)}^{*}$ CIs is geometrically close to the $M_{\pi \pi^{*}}$ structure.

Table 4 compiles the energy of six CIs geometries (*a*1–*a*3 and *b*1–*b*3) obtained by linear interpolation (LIP) between the $M_{\pi \pi^{*}}$ and the $M_{n\pi_{\left(II\right)}^{*}}$ minima. In addition critical CI points, such as minimum energy CI (MECI) and minimum distance CI (MDCI) from $M_{\pi \pi^{*}}$, are given [36]. All geometries belong to the same $\pi \pi^{*}$/$n\pi_{\left(II\right)}^{*}$ CI seam. In NAPA B, two MECI structures differing in the direction of the N atom pyramidalization ($\vartheta_{N}$) are located 0.13 and 0.16 eV above $M_{\pi \pi^{*}}$. In NAPMA B, two analogous, but isoenergetic structures are found 0.17 eV above $M_{\pi \pi^{*}}$. The two MDCI structures, which are expected to represent a dynamically important portion of the CI seam, are located 0.22 eV and 0.28 eV above $M_{\pi \pi^{*}}$ in NAPA B and NAPMA B, respectively. Comparing the MDCI and $M_{\pi \pi^{*}}$ structures, a major difference is found only in the C=O bond elongation. Altogether, the structural analysis reveals that NAPA B and NAPMA B share a quite similar topography of the $\pi \pi^{*}$/$n\pi_{\left(II\right)}^{*}$ CI seam.

In terms of energy, one sees that as a consequence of smaller RMSD from $M_{\pi \pi^{*}}$, the barriers in the basin *a* are lower than the ones in *b*. Furthermore, the barriers to equivalent CI geometries are systematically lower in NAPA B than in NAPMA B. In particular, the lowest barriers corresponding to MECI*a*, CI*a*1 and CI*a*2 increase from 0.13, 0.22 and 0.19 eV in NAPA B to 0.17, 0.32 and 0.25 eV in NAPMA B. However, these differences are not large enough to exclude the possibility of NAPMA B deactivation by Mechanism II.

**Figure 7:**
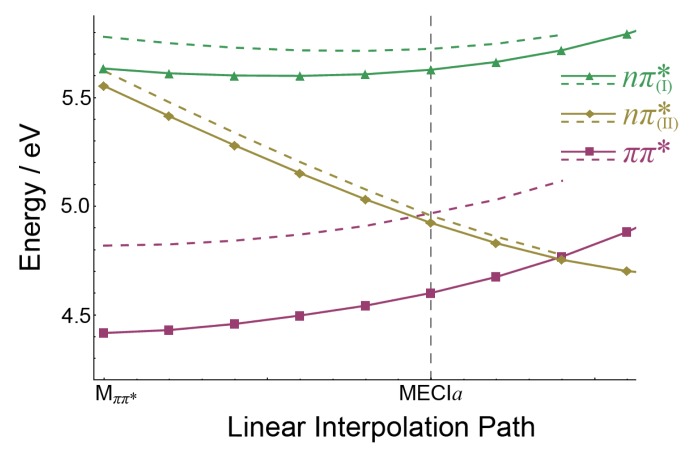
LIP constructed between the $M_{\pi \pi^{*}}$ and MECI*a* geometries computed at the RI-CC2/cc-pVDZ (dashed lines) and CASPT2/cc-pVDZ (solid lines) levels of theory. The $\pi \pi^{*}$ (bordeaux), $n\pi_{\left(II\right)}^{*}$ (gold) and $n\pi_{\left(I\right)}^{*}$ (green) adiabatic states are indicated. MS-CASPT2/cc-pVDZ excitation energies are computed at RI-CC2/cc-pVDZ geometries.

Before addressing the problem of the accessibility of the $\pi \pi^{*}$/$n\pi_{\left(II\right)}^{*}$ CI seam, it is important to verify that the CC2 method describes adequately this region of the configuration space. Figure 7 compares CASPT2 (solid lines) and CC2 (dashed lines) results for an LIP constructed between the $M_{\pi \pi^{*}}$ and MECI*a* geometries. Both $M_{\pi \pi^{*}}$ and MECI*a* geometries have been optimized at the CC2/cc-pVDZ level. The energy profiles of the $\pi \pi^{*}$, $n\pi_{\left(II\right)}^{*}$ and $n\pi_{\left(I\right)}^{*}$ states are shown. It is evident that CC2 and CASPT2 LIPs follow the same trend. However, the CC2 method overestimates the energy of the $\pi \pi^{*}$ state by 0.4 eV (see also Table 2) and consequently underestimates the barrier to the $\pi \pi^{*}$/$n\pi_{\left(II\right)}^{*}$ CI. This means that the problem of the accessibility of the $\pi \pi^{*}$/$n\pi_{\left(II\right)}^{*}$ seam is actually more acute at the CASPT2 level and needs to be carefully investigated. We will do this in the next section.

### 3.4. Accessibility of the $\pi \pi^{*}$/$n\pi_{\left(II\right)}^{*}$ Seam of CI in NAPA B and NAPMA B

To investigate the depopulation of the vibrationless phenyl $\pi \pi^{*}$ state, it is better to switch to the diabatic representation in which a $\pi \pi^{*}$ state nuclear wave packet can be examined as a standing wave function in the minimum well of the $\pi \pi^{*}$ PES. By neglecting the nuclear kinetic energy term, the time-dependent Schrödinger equation for an arbitrary number of states in diabatic representation can be easily transformed to the following form:

(1) ∂Pππ*(r,t)∂t≈2Im∑j≠ππ*Wππ*,j(r)Ψ¯ππ*(r,t)Ψj(r,t),

which describes the probability density $P_{\pi \pi^{*}}\left(r,t\right)={\bar{\Psi }}_{\pi \pi^{*}}\left(r,t\right)\Psi_{\pi \pi^{*}}\left(r,t\right)$ of the initial $\pi \pi^{*}$ state in terms of $\Psi_{\pi \pi^{*}}$ and $\Psi_{j}$ diabatic nuclear functions of $\pi \pi^{*}$ and remaining *j* states, respectively. The bar designates complex conjugation, and $r=(r_{1},\ldots ,r_{N})$ denotes the Cartesian coordinate vector with components $r_{i}=\left(r_{i,x},r_{i,y},r_{i,z}\right)$. $W_{\pi \pi^{*},j}$ are the diabatic coupling terms describing the interaction strength between $\pi \pi^{*}$ and *j* electronic states and, as the adiabatic terms, are most relevant around CI points. However, contrary to adiabatic, the diabatic couplings vanish at CI points. By integrating the above expression over the entire nuclear configuration space, the time-dependent population of the $\pi \pi^{*}$ state is obtained:

(2) ∂Pππ*(t)∂t≈2∑j≠ππ*∫ππ*/jCIspaceWππ*,j(r)ImΨ¯ππ*(r,t)Ψj(r,t)dr.

On the right-hand side, the integration is restricted only to the portion of the configuration space around the CI seams where the couplings do not vanish. Because the $\Psi_{j}$ values are proportional to the $\Psi_{\pi \pi^{*}}$ wave function values in the diabatic Schrödinger equation, the expression reduces to:

(3) ∂Pππ*(t)∂t≈∑j≠ππ*∫ππ*/jCIspaceW˜ππ*,j(r)Ψ¯ππ*(r,t)Ψππ*(r,t)dr.

By approximating the wave functions as the product of their space and time independent parts, Ψ(r,t)≈Ψ(r)Φ(t)⇔P(r,t)≈ρ(r)P(t), the previous expression further simplifies to:

(4) ∂Pππ*(t)∂t≈Pππ*(t)∑j≠ππ*∫ππ*/jCIspaceW˜ππ*,j(r)ρππ*(r)dr,

with only the spatial part of the $\pi \pi^{*}$ wave function left under the integral. This shows that the time-dependent population of the $\pi \pi^{*}$ state satisfies the first order rate law with the above integrals as the rate constants $\kappa_{\pi \pi^{*}\to j}:$

(5) $$\frac{\partial P_{\pi \pi^{*}}\left(t\right)}{\partial t}\approx -\sum_{j\neq \pi \pi^{*}}\kappa_{\pi \pi^{*}\to j}P_{\pi \pi^{*}}\left(t\right).$$

The obtained first order rate is consistent with the monoexponential decay of the phenyl $\pi \pi^{*}$ first excited state in NAPA and NAPMA conformers as observed in the experiments [17,18]. As stated, the decay rate depends on the two terms, the coupling factor between the electronic states and the probability $\rho_{\pi \pi^{*}}\left(r\right)$ of the starting $\pi \pi^{*}$ state to access the interaction region. Unfortunately, for the systems under investigation, the coupling terms are not available. However, owing to the similarity of the electronic states, large differences in the couplings cannot be assumed. Furthermore, the distinctive sharp crossings obtained between $\pi \pi^{*}$ and $n\pi_{\left(II\right)}^{*}$ PES indicates that they are small and localized around CI points, which further emphasize the importance of the accessibility of the nuclear wave function to the corresponding CI seam.

Let us remind that lifetimes of the three NAPA/NAPMA conformers were measured at the origin of the S_1_ $\left(\pi \pi^{*}\right)$ state (see also Scheme 2). Because of vibrationless excitation, the ZPE is the only energy available to the system. Therefore, we need to estimate whether a given CI geometry is classically-accessible or not to the vibrationless nuclear wave function. We retain only the harmonic terms in the normal mode expansion of the $\pi \pi^{*}$ PES and compute the extension of the normal mode coordinates at the CI geometries. The Cartesian coordinate vector of the CI geometry is denoted as r and the mass-weighted Cartesian coordinate as R with $R_{i,\alpha }=r_{i,\alpha }\sqrt{m_{i}}$, α=x,y,z. The normal mode vector Q is given in terms of mass-weighted Cartesian displacements from a reference geometry $R^{ref}$ as:

(6) $$Q=L(R-R^{ref})$$

where L is the unitary transformation matrix containing the eigenvectors of the Hessian matrix evaluated at the reference geometry $R^{ref}$. In our case, the reference geometry is $M_{\pi \pi^{*}}$, i.e., the initially excited geometry. The full dimensional harmonic $\pi \pi^{*}$ PES is then given as:

(7) $$W\left(Q\right)=\frac{1}{2}\sum_{i=1}^{3N-6}\omega_{i}^{2}Q_{i}^{2}$$

where $\omega_{i}$ is the frequency of the *i*-th normal mode. In principle, the quality of the harmonic approximation is questionable, but in both NAPA and NAPMA, it was found adequate, at least for the purpose of our investigation. This can be seen from the relatively small differences between the exact (LIP) and approximate (HAR) energies of the CI geometries given in Table 4.

The potential energy along each normal mode coordinate evaluated at the CI geometry ($W(Q_{CI,i})$) can now be compared to the ZPE and the ratio (atomic units):

(8) $$\frac{W_{i}\left(Q_{CI,i}\right)}{ZPE_{i}}=\frac{\frac{1}{2}\omega_{i}^{2}Q_{CI,i}^{2}}{\frac{1}{2}\omega_{i}}\left\{\begin{array}{c} \leq 1\;;classically\;accessible\\ >1\;;classically\;inaccessible \end{array}\right.$$

**Figure 8:**
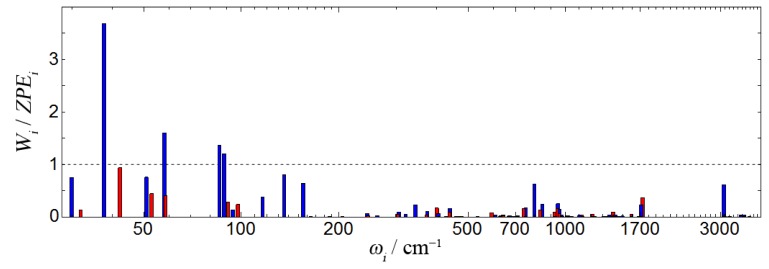
Classical accessibility to the MECI*a* geometry for NAPA B (red) and NAPMA B (blue). For each normal mode, the ratio between the normal mode’s potential energy $W_{i}=\omega_{i}^{2}Q_{CI,i}^{2}/2$ and its zero point energy, $ZPE_{i}=\omega_{i}/2$, is shown. A ratio $W_{i}\left(Q_{i}\right)/ZPE_{i}<1$ for all normal modes indicates a classically-accessible CI.

This determines whether the normal mode coordinate $Q_{i}$ lies in a classically-accessible or inaccessible part of the configuration space. It is important to stress that in order to be classically-accessible, a CI geometry needs to be accessible along all normal modes. A representative example is given in Figure 8 where the MECI*a* geometry is analyzed in terms of the $W_{i}\left(Q_{CI,i}\right)/ZPE_{i}$ ratio. It is apparent that in NAPA B (red), the MECI*a* geometry is classically-accessible along all of the modes as the ration $W_{i}\left(Q_{CI,i}\right)/ZPE_{i}<1$. In contrast, four low frequency normal modes of NAPMA (blue) lie in the classically inaccessible region. In the same fashion, we assess the accessibility of the key CI geometries compiled in Table 4. Those that are classically-accessible are given in bold.

The question that naturally arises is what are the consequences of the reduced accessibility of some CI in NAPMA. The probability to reach the CI seam and the rate of decay of the $\pi \pi^{*}$ state are clearly connected. Semiclassically, when $W_{i}\left(Q_{i}\right)/ZPE_{i}>1$ for one of the normal modes, the probability, $P_{i}$, to reach the CI geometry every time the particle “hits” the classical turning point, $Q_{0,i}$, is [37]:

(9) $$P_{i}={\left|\Psi \left(Q_{CI,i}\right)\right|}^{2}=e^{2S}$$

where *S* is the action integral through the barrier:

(10) $$S=-\int_{Q_{i,0}}^{Q_{CI,i}}{\dot{Q}}_{i}dQ_{i}=-\int_{Q_{0,i}}^{Q_{CI,i}}\sqrt{\omega_{i}^{2}Q_{i}^{2}/2-\omega_{i}/2}\;dQ_{i}=-\int_{Q_{0,i}}^{Q_{CI,i}}\sqrt{W_{i}-ZPE_{i}}\;dQ_{i}.$$

Expressing the integral in terms of $\alpha_{i}=W_{i}/ZPE_{i}$, one easily obtains the probability that the system reaches the CI along the *i*-th normal mode as:

(11) $$P_{i}\left(\alpha_{i}\right)=e^{-\alpha_{i}\sqrt{\alpha_{i}^{2}-1}}\left(\alpha_{i}+\sqrt{\alpha_{i}^{2}-1}\right)$$

For *n* modes in the tunneling regime, the total probability to reach the CI is the product of probabilities along individual modes, $P=\prod_{i=1}^{n}P_{i}$.

Let us return to the example of Figure 8. If we assume that the probability to reach MECI*a* in NAPA B is P=1, then the probability to reach MECI*a* in NAPMA is only P=0.10. In the case of the *a*1 CI geometry, it is P=0.15. Of course, these are very rough approximations for the 32-times increase of the lifetime of NAPMA with respect to NAPA, but they illustrate how an energy difference of only 0.04 eV (MECI*a*) can lead to a 10-fold reduction of the probability of internal conversion.

**Figure 9:**
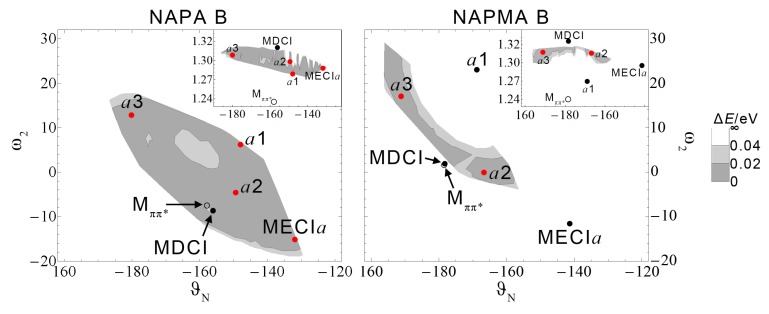
Two-dimensional projections of the classically-accessible portion of the $\pi \pi^{*}$/$n\pi_{\left(II\right)}^{*}$ seam in the basin *a* of NAPA (left) and NAPMA (right). Projections on the $\vartheta_{N}$ and $\omega_{2}$ angles are show in the main panel. Insets show the same portion of the seam projected on the subspace spanned by $d(C_{Phe}=O)$ (vertical axis, in Å) and $\vartheta_{N}$ (horizontal axis, in degrees). In total, 153 NAPA and 63 NAPMA CI geometries have been used. The reference zero point energy (ZPE) accessible (inaccessible) LIP/MECI structures are shown with red (black) dots. Open circles indicate $M_{\pi \pi^{*}}$. Shades of gray indicate the size of the energy gap (eV) between the two adiabatic states.

Owing to the high dimensionality of the seam space, it is desirable to go beyond the analysis of individual CI geometries. A glimpse into the reduction of the classically-accessible volume of the CI seam space in NAPMA can be obtained by considering two-dimensional (2D) projections of the seam space on the torsional angles $\vartheta_{N}$ and $\omega_{2}$ and the *d*(C=O) stretching coordinate. In total, 153 NAPA and 63 NAPMA CI geometries have been generated first by interpolating between pairs and triplets of reference CI geometries (Table 4) and then by including in the search the newly-obtained CI geometries that satisfied the ZPE accessibility condition. Figure 9 shows the 2D projections of the classically-accessible portion of $\pi \pi^{*}$/$n\pi_{\left(II\right)}^{*}$ CI seams in NAPA B (left) and NAPMA B (right). ZPE-accessible areas are shown in gray with the two shades indicating the size of the energy gap, i.e., the accuracy of the calculation.

The difference between the classically-accessible portion of the seam in NAPA B and NAPMA B is stunning. The characteristics of the CI geometries given in Table 4 are captured in Figure 9. In NAPA B, MECI*a* and *a*1 are on the edge of the classically-accessible area, while *a*2 and *a*3 CI are deep in the ZPE-accessible region. For NAPMA, MECI*a* and *a*1 are far from the classically-accessible area.

Altogether, we found that the NH_2_ group of NAPA B, already distorted in the $M_{\pi \pi^{*}}$ minimum, requires a small extension of the torsional normal modes to reach the CI seam. In contrast, the second amide group in NAPMA B is planar in $M_{\pi \pi^{*}}$, and the extension of several low frequency normal modes beyond the classical turning point is needed to reach the CI seam. The classically-accessible portion of the NAPMA CI seam is reduced, and the decay of the $\pi \pi^{*}$ state is slower.

### 3.5. Alternative Nonradiative Deactivation Pathways: Phenyl Ring Puckering and Intersystem Crossing

**Figure 10:**
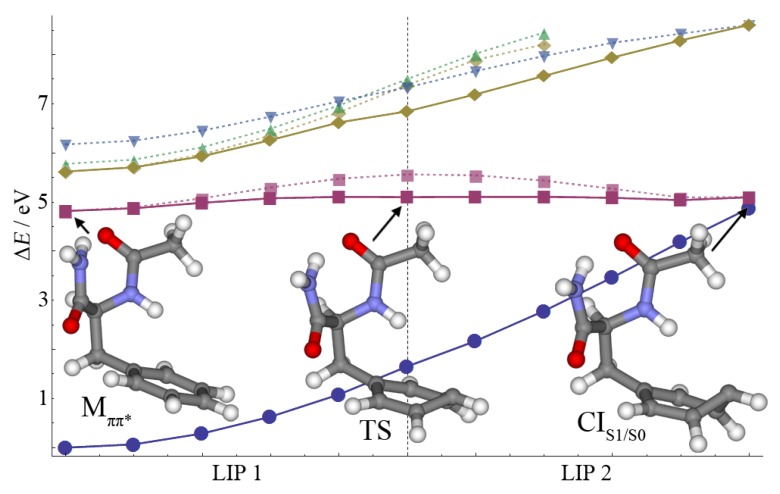
Energy profiles of the two lowest excited states and the electronic ground state for phenyl ring-puckering at the *para* C atom position in NAPA B. The insets show the $M_{\pi \pi^{*}}$ minimum, the S_1_ transition state (TS) and the S_1_/S_0_ CI structure. The TS structure was obtained by reoptimizing the starting maximum energy geometry taken from the initial LIP between $M_{\pi \pi^{*}}$ and CI geometry (bordeaux dashed line). Two separate LIPs between $M_{\pi \pi^{*}}$ minimum and the TS (LIP1) and between TS and CI (LIP2) are fused to obtain the full reaction profile.

The ring-puckering mechanism of benzene [38] was reconstructed in NAPA B to investigate the possibility of direct deactivation from the $\pi \pi^{*}$ state to the electronic ground state. First, the phenyl moiety was isolated and optimized to the lowest prefulvene symmetrical MECI between the S_1_ and S_0_ states using the method of Levine et al. [36] starting from structural parameters from [38]. Since six distinctive positions and two puckering direction are possible in the bounded phenyl ring of NAPA, only three sites, *ortho*, *meta* and *para*, closest and in the direction of the N_*Phe*_–H bond were investigated. The puckered benzene ring was then attached to the peptide backbone and the system reoptimized to obtained the new S_1_/S_0_ MECI structure. We found that structurally, the puckered phenyl ring in NAPA B does not deviate much from the starting benzene CI structure regardless of the puckering position. The LIPs constructed between CIs and the $M_{\pi \pi^{*}}$ minimum revealed barriers of 0.65, 0.63 and 0.74 eV for puckering at the *ortho*, *meta* and *para* positions, respectively. Since the estimated barriers are larger than the reported values for benzene [38,39], we optimized the S_1_ transition states (TS). Figure 10 displays the obtained TS structure together with the MECI geometry along the ring-puckering pathway at the *para* C position. The vibrational analysis confirmed one imaginary frequency in the TS structure. In all three ring-puckering mechanisms, TSs were found 0.28 eV above the $M_{\pi \pi^{*}}$ minimum, which is ∼0.1–0.2 eV lower than the MC-SCF values for benzene [38,39]. Apart from the difference in barrier height, no significant effects of the backbone on the ring-puckering mechanisms were observed. Altogether, we found that all three ring-puckering pathways are classically inaccessible from the vibrationless states of NAPA B and NAPMA B, as was actually expected from previous theoretical studies of benzene [38,39,40,41,42,43].

Next, the contribution of the intersystem crossing (ISC) is considered. ISC is the main deactivation channel of vibrationless $\pi \pi^{*}$ states of benzene and toluene. Specifically, the lifetime of toluene is 86.4 ns [31,44], which is very similar to the lifetime of NAPA and NAPMA conformers A, as well as of NAPMA conformer C (see Table 1). These observations agree with the El-Sayed result that the ISC rate for singlet $\pi \pi^{*}$ to triplet $n\pi^{*}$ state transfer between phenyl and N atom containing groups might be on the order of a few tens of ns [45]. However, the 1.5-ns lifetime of NAPA B is significantly shorter.

**Figure 11:**
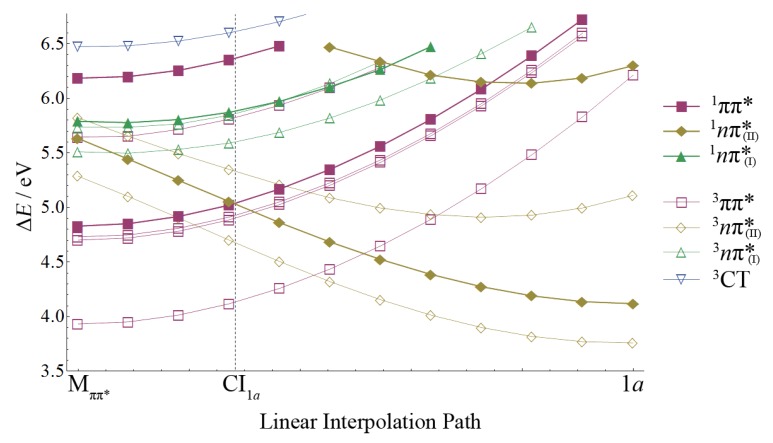
LIP constructed between $M_{\pi \pi^{*}}$ and M_*a*1_ geometries. Singlet (triplet) excited states are shown with filled (unfilled) symbols. The vertical line indicates the position of the ^1^$\pi \pi^{*}$/^1^$n\pi_{\left(\mathrm{II}\right)}^{*}$ CI. Adiabatic states are color coded according to their characters.

To include the triplet states in the description of NRD mechanisms of NAPA and NAPMA, spin coupling terms between states of different multiplicity would be required. Unfortunately, these are unavailable at the CC2 level of theory. Therefore, we compared only the energy profiles of the triplet and singlet states. Figure 11 shows the LIP between the $M_{\pi \pi^{*}}$ and M_*a*1_ minimum of NAPA B. Low lying singlet and triplet states are indicated with filled and unfilled symbols, respectively. Focusing on the triplet ^3^$n\pi_{\left(\mathrm{II}\right)}^{*}$ state, one sees that it intersects with the phenyl singlet ^1^$\pi \pi^{*}$ prior to the singlet ^1^$n\pi_{\left(\mathrm{II}\right)}^{*}$ PES. The ^1^$\pi \pi^{*}$/^3^$n\pi_{\left(\mathrm{II}\right)}^{*}$ crossing is found barely 0.01 eV above the $M_{\pi \pi^{*}}$ minimum and is classically-accessible, indicating a potential contribution of ISC to NAPA B deactivation. However, this observation should be taken with caution. Namely, due to the more pronounced stability of triplet states, most of the ^1^$\pi \pi^{*}$/^3^$n\pi_{\left(\mathrm{II}\right)}^{*}$ crossing seam is classically-accessible in all NAPA and NAPMA conformers. As no difference in the spin-orbit couplings between structurally equivalent NAPA and NAPMA conformers should be a priori anticipated, the ISC mechanism actually fails to explain the experimental difference in the lifetimes of NAPA B and NAPMA B. Therefore, Mechanism II remains the more plausible deactivation pathway of NAPA and NAPMA conformer B.

### 3.6. Investigation of Nonradiative Deactivation Pathways in NAPA Monohydrate

**Figure 12:**
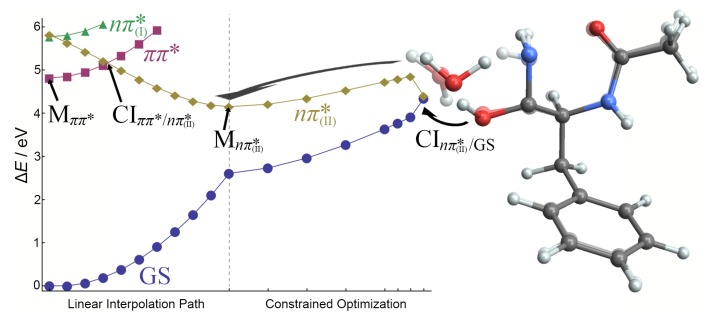
Energy profile showing the deactivation of NAPA B monohydrate. In the first part, the reaction path was obtained by linear interpolation between the $M_{\pi \pi^{*}}$ and M_(*II*)_ minima (left of the dashed line). In the second part, the energy profiles were obtained by constrained optimization of the $n\pi_{\left(II\right)}^{*}$ state for fixed $O_{H_{2}O}-$H bond distances.

In NAPAH B, the monohydrate of NAPA conformer B, water is H-bonded to the second amide group. On the basis of the results obtained for NAPA and NAPMA, we concentrate on the $n\pi_{\left(II\right)}^{*}$ state. From the vertical excitation energies reported in Table 2, it is evident that hydration destabilizes the $n\pi_{\left(II\right)}^{*}$ state. At the geometry of vertical excitation, the energy gap between the $n\pi_{\left(II\right)}^{*}$ and the $\pi \pi^{*}$ states increased from 0.43 (0.52) eV in NAPA (NAPMA) to 0.635 in NAPAH. Thus, unless a new deactivation mechanism sets in, NAPAH should relax more slowly than NAPA and NAPMA. Upon geometry optimization of the $n\pi_{\left(II\right)}^{*}$ state, several minima have been found. A one to one comparison between NAPA and NAPAH minima is, however, not possible. On the one hand, not all NAPA (NAPMA) minima are realizable in NAPAH, as some cannot accommodate a hydrogen-bonded water molecule. On the other hand, NAPAH M_(*II*)_ minima are characterized by the high flexibility of the water molecule. Water can occupy a range of positions independently of the distortion of the second amide group, meaning that for a given NAPA minimum, one finds several M_(*II*)_ minima in NAPAH. Figure 12 shows the most stable NAPAH B M_(*II*)_ minimum, which is also geometrically the closest to the starting $M_{\pi \pi^{*}}$ structure. In this particular M_(*II*)_ minimum, the water molecule is attached with two hydrogen bonds to the second amide group, but the co-linearity of the O–H···O_(*II*)_ hydrogen bond, and thus, its strength, is reduced due to the decreased electronic density on the O_(*II*)_ atom.

To understand how microsolvation affects the deactivation of the $n\pi_{\left(II\right)}^{*}$ state, we investigated two possible relaxation pathways. Both start from the $M_{\pi \pi^{*}}$ minimum and reach one of the M_(*II*)_ minima, but differ in the mechanism of $n\pi_{\left(II\right)}^{*}$ deactivation.

In the first, shown in Figure 12, the reaction coordinate is the elongation of the $O_{H_{2}O}$–H bond in the $O_{H_{2}O}$–H···O_(*II*)_ moiety. It is evident that $O_{H_{2}O}$–H bond elongation leads to a continuum increase of the energies of S_0_ and S_1_ states. After the double hydrogen transfer, a sudden decrease of the energy of the $n\pi_{\left(II\right)}^{*}$ state resulted in a CI that intersects with the ground state at a point almost isoenergetic to the $M_{\pi \pi^{*}}$ minimum. A reaction barrier of 0.7 eV is found on the H-transfer path. At the CI geometry, whose energy slightly exceeds the energy of the $M_{\pi \pi^{*}}$ minimum, the hydrogen atom is transferred to the O_(*II*)_ atom, and the distal H atom of the amino group is transferred to water to compensate for the missing H atom. The intersection geometry can be viewed as a highly deformed structure of the imidic acid form of the second amide group. The changes in other bonds are very small, below 0.02 Å.

**Figure 13:**
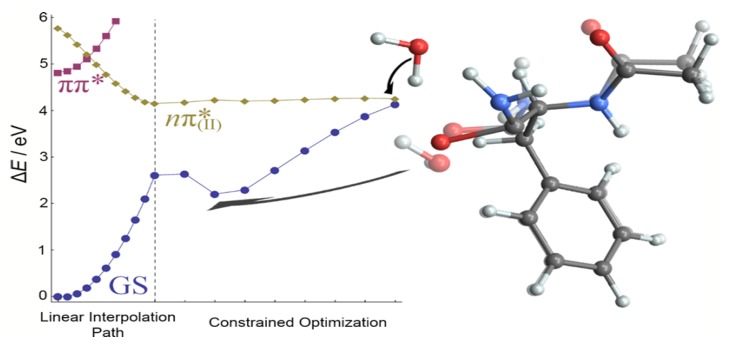
Energy profile showing the deactivation of NAPA B monohydrate. In the first part, interpolation was performed between the $M_{\pi \pi^{*}}$ and M_(*II*)_ minima (left of the dashed line). In the second part, the energy profiles were obtained by constrained optimization in the $n\pi_{\left(II\right)}^{*}$ state for fixed C_*Phe*_=O bond distances.

The second mechanism is shown in Figure 13. Here, in analogy to the deactivation of NAPA and NAPMA, we elongated the C_*Phe*_=O bond. One immediately sees that the extension of the C_*Phe*_=O bond leads only to a marginal increase in the energy of the $n\pi_{\left(II\right)}^{*}$ state. However, the geometrical changes along the path are significant as the two overlapped geometries clearly show. Already a 0.16 Å extension of the C_*Phe*_=O induces a major change in the structure of the monohydrate as water repositions to preserve the two hydrogen bonds. To reach the CI with the ground state, the C_*Phe*_=O bond needs to be extended by 0.48 Å. The RMSD between the intersection and the M_(*II*)_ minimum structures is 2 Å while in the hydrogen transfer mechanism, the RMSD is ten-times smaller. However, the system needs to preserve both hydrogen bonds in order to deactivate via hydrogen transfer, whereas C_*Phe*_=O pre-dissociation can potentially proceed from any $n\pi_{\left(II\right)}^{*}$ structure.

To explore the initial steps of NAPAH B deactivation on the $n\pi_{\left(II\right)}^{*}$ surface, we have launched 10, 350 fs-long, RI-CC2/cc-pVDZ trajectories from the $\pi \pi^{*}$/$n\pi_{\left(II\right)}^{*}$ MECI. The initial velocities were obtained from the Wigner distribution function for the harmonic ZPE modes of the $M_{\pi \pi^{*}}$ minimum evaluated at the MECI geometry. Because of the initial C=O bond extension, the potential energy of the ground state rose very sharply, reducing the gap between S_1_ and S_0_ to ∼1.0 eV. After a few fs, the energy of S_2_ increased, and the state remained well separated from S_1_ for the whole duration of the propagation. The water-amino group hydrogen bond has been preserved in all trajectories. In contrast, the hydrogen bond with the C=O oxygen was easily disrupted. A twisting motion around the $O_{H_{2}O}\cdot \cdot \cdot$H−N_(*II*)_ hydrogen bond was observed, but we have found no evidence of significant vibrational excitations of the water O−H bonds. Within the short propagation period, only one trajectory reached the S_1_/S_0_ CI. Compared to $M_{\pi \pi^{*}}$, in the CI geometry, the C=O bond was elongated by more than 0.55 Å. Altogether, combined reaction path calculations and dynamics simulations show that once in the $n\pi_{\left(II\right)}^{*}$ state, NAPAH deactivates by C_*Phe*_=O bond extension. We have found no evidence that intermolecular H-transfer may speed up the deactivation of the $n\pi_{\left(II\right)}^{*}$ state.

In analogy to NAPA B and NAPMA B, the key step in the deactivation of NAPA monohydrates is the transfer of excitation from the $\pi \pi^{*}$ to the $n\pi_{\left(II\right)}^{*}$ state. Following the procedure detailed in previous sections, a set of $\pi \pi^{*}$/$n\pi_{\left(II\right)}^{*}$ CI geometries was constructed by linear interpolation between the $M_{\pi \pi^{*}}$ and different M_(*II*)_ minima. Since CI geometries are linear combinations of two minima, their geometrical changes relative to the starting $M_{\pi \pi^{*}}$ minimum are a fraction of the difference between the two minima. However, all CI geometries are structurally closer to $M_{\pi \pi^{*}}$. Among the nine CI geometries, six belonged to the *a* class of CI structures and the remaining three to class *b*. In both sets, CI geometries are at least 0.27 eV higher than the $M_{\pi \pi^{*}}$ structure. Compared to NAPA and NAPMA CI structures, they display a smaller energy variation (0.29 ± 0.02 eV). The constructed CI geometries have been used to obtain MECIs. Table 5 compiles the relative energies of four MECI geometries, one of the MECI*b* type (see Table 3 and Table 4) and three of the MECI*a* type. All four are found higher in energy than their NAPA and NAPMA counterparts. MECI*a*1 and MECI*a*3 differ in the position of the water molecule, which changes from one side of the plane defined by the second amide group to the other with a difference of 25° in the torsional angle $\omega_{H_{2}O}$ ($O_{H_{2}O}$−N−C−O). This indicates that the CI seam topography may be rather flat with respect to the water motion.

**Figure 14:**
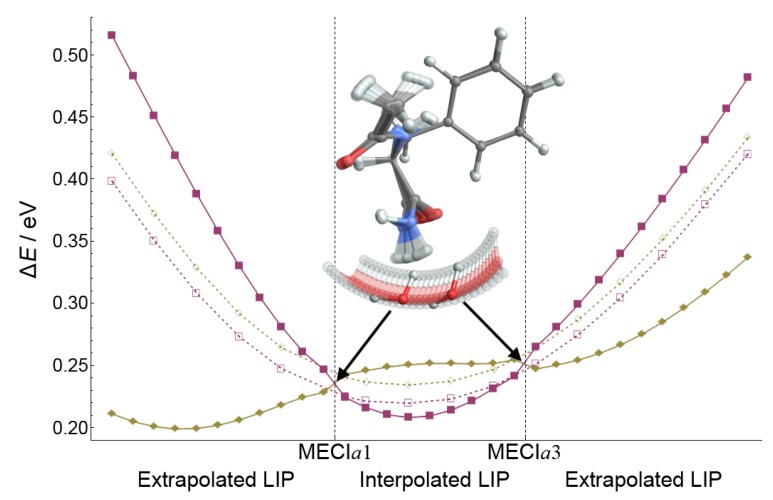
Splitting of degeneracy between $\pi \pi^{*}$ (bordeaux) and $n\pi_{\left(II\right)}^{*}$ (gold) in the region around MECI*a*1 and MECI*a*3. The geometries on the interpolated and extrapolated part of the reaction path are shown in the inset. Energy profile (eV) of the reference CI seam (dashed).

The topography of the CI seam was investigated by interpolating the structures between two MECI*a* geometries, as well as extrapolating from them. As shown with full lines in Figure 14, motion along the LIP coordinate causes a degeneracy breaking between the electronic states, even in the interpolated region between the two MECIs. Apart from the huge change in the position and orientation of water, the second amide group exhibits a change of the $\omega_{2}$ torsion angle by 7° from one MECI to the other. The remaining covalent bonds change by less than 0.2%.

Dashed lines show the energy changes of the true CI seam. The geometries on the CI seam have been obtained as minimum distance CI geometries from the linearly interpolated/extrapolated geometries. From the change in the energy profile of the $\pi \pi^{*}$ state (bordeaux) and of the seam (dashed line), it is evident that the $\pi \pi^{*}$ state is the one strongly affected by the disruption of the hydrogen bonds. While the $n\pi_{\left(II\right)}^{*}$ state is only marginally affected, the energy of the $\pi \pi^{*}$ state changes rapidly, causing the splitting of the two PESs. Thus, coordinates describing the position and orientation of water with respect to the second amide group contribute to the branching coordinates.

The results obtained for the NAPA monohydrate fit well within the broader picture of peptide deactivation in which the flexibility of the second amide group promotes the deactivation. We have shown that the remarkable flexibility of the terminal NH_2_ group is the cause of the faster deactivation of NAPA with respect to NAPMA. In the monohydrate, the NH_2_ flexibility is hampered by two hydrogen bonds preventing efficient transfer from the Phe side chain to the peptide backbone.

## 4. Methods

The ground state structures of all experimentally-observed NAPA, NAPMA and NAPAH conformers, and the corresponding Hessian matrices were computed at the CC2 level using the resolution of identity (RI) approximation [12,13]. The cc-pVDZ basis set is used throughout the work [46]. The absorption spectra were obtained with the RI-CC2 and complete-active-space second-order perturbation theory (CASPT2) [47,48,49]. The adiabatic excitation energies and excited state Hessians were computed only at the RI-CC2 level. The reference CASSCF orbitals were obtained in the state-averaged mode, with an active space consisting of 14 electrons in 12 orbitals. Six benzene *π* type orbitals (a_2*u*_, e_1*g*_, e_2*u*_, $e_{1g}^{*}$, $e_{2u}^{*}$, $b_{1g}^{*}$) localized on the phenyl ring, a pair of *π* and $\pi^{*}$ on each C=O group and one nonbonding *n* orbital on each oxygen atom, giving the minimum orbital set needed to accurately describe the electronic transitions. The same CASSCF/CASPT2 settings were used as in previous work [27].

The competition between different deactivation mechanisms active in NAPA conformer B were studied by nonadiabatic (NA) dynamics simulations with fewest switches trajectory surface hopping [50] using an in-house software [17,51,52,53]. The simulations were based on TDDFT (PBE0) electronic structure computations. The initial conditions were sampled randomly from a ground state trajectory at T=298 K. From the experiment, it is known that the estimated internal beam temperature is <10 K. However, taking into account the 1.5 ns-long excited state lifetime of NAPA B, it was deemed necessary to run room temperature simulations in order to facilitate the sampling of different deactivation mechanisms over a reasonable period of time [17]. Furthermore, since the energy difference between TD-PBE0 excited states is smaller than the one between RI-CC2 states, this additionally facilitated switches between the surfaces. The velocity Verlet algorithm with a time step of Δt=0.25 fs was used to integrate the nuclear equations of motion. The NA-TDDFT trajectories were propagated in a subspace spanned by the ground and the four lowest excited electronic states. All trajectories were initiated from the first excited state and followed for 1250 fs. Because the TDDFT method cannot describe the S_1_/S_0_ CI topology properly, the last point of the trajectory is merely taken as the indication that a real S_1_/S_0_ CI point is located nearby. Alternatively, a threshold value of 0.1 eV for the S_1_-S_0_ energy gap can be used as an indication of a CI [54], but this criterion has not been used in this work. For more information on the NA dynamics simulations of NAPA B, see [17].

NA dynamics simulations of NAPAH conformer B were performed using the RI-CC2 method [55,56,57]. Here, a different strategy has been employed to incite the deactivation. The trajectories were started in the S_1_ state with the geometry of the minimum energy CI (MECI) between the $\pi \pi^{*}$ and $n\pi_{\left(II\right)}^{*}$ states and propagated in the manifold of the ground and three excited states for a maximal time of 350 fs. Velocities were selected randomly from from a harmonic Wigner distribution function evaluated at the starting MECI geometry. Nonadiabatic couplings were not computed, as the S_1_ and S_2_ states were well separated after leaving the CI region.

The main deactivation pathways, mostly indicated by dynamics simulations, were reinvestigated by reaction path calculations. Linear interpolation in internal coordinates has been used to connect the corresponding minima of the initially excited S_1_($\pi \pi^{*}$) state with a number of minima located on the $n\pi_{\left(II\right)}^{*}$ surface. MECI geometries have been located using the sequential penalty constrained optimization method of Levine et al [36]. MOLCAS, Version 6.4, was used for the CASSCF/CASPT2 and TURBOMOLE, Versions 6.4 and 7.0, for the RI-CC2 and TD-PBE0 calculations [58,59].

## 5. Concluding Remarks

This work compares the photodynamics of three neutral model peptides that takes place after optical excitation of the phenylalanine residue. The $\gamma_{L}\left(g+\right)$ folded form of *N*-acetylphenylalaninylamide (NAPA B), its amide-N-methylated derivative (NAPMA B) and the monohydrate (NAPAH B) were investigated with the goal of unraveling how chemical substitution impacts the photodynamics. Namely, lifetime measurements at the origin of the first $\pi \pi^{*}$ state have revealed a remarkably short lifetime of NAPA B. Under the same excitation conditions, the lifetime of NAPMA B was found to be 30-times longer.

We have shown that excitation transfer from phenyl $\pi \pi^{*}$ to amide $n\pi^{*}$ states plays a central role in the deactivation of the three peptides and that, once in the $n\pi^{*}$ state, several barrierless deactivation pathways become possible. Moreover, we have shown that the ease of the amide group out-of-plane distortions determines the rate of population transfer from the minimum of the $\pi \pi^{*}$ to the $n\pi^{*}$ state. Specifically, the enhanced rigidity of the terminal amide group induced by methylation in NAPMA and hydrogen bonding in NAPAH leads to significant reduction of the classically-accessible portion of the CI seam from the photoexcited state. As vibrational excitation of several low-frequency normal modes is needed in order to cross the barrier to the $\pi \pi^{*}$/$n\pi^{*}$ CI, excitation at the origin of the $\pi \pi^{*}$ transition results in excited state lifetimes in the nanosecond timescale.

Generalization to larger peptides or proteins is difficult. However, we anticipate that apart from the N-terminal primary amide groups, flexible amide group in the side chain of glutamine or asparagine could also act as quenchers of excitation. The excitation transfer from the side chain of the chromophoric amino acid to the peptide backbone critically depends on the accessibility of the $\pi \pi^{*}$/$n\pi^{*}$ CI seam. Therefore, vibrational excitation may lead to faster nonradiative deactivation as has been recently shown for Ac-Gly-Phe-NH_2_ [19].

## Figures and Tables

**Table 1:** Excited state lifetimes (in ns) of selected isotopomers of NAPA and NAPMA measured at the origin of the $\pi \pi^{*}$ transition by pump-probe experiments [17,18].

	A	B	C
NAPA	70 ± 2	1.5 ± 0.3	35 ± 2
NAPMA	67 ± 3	48 ± 3	62 ± 3

**Table 2:** Vertical absorption energies to the four lowest excited singlet states of conformers NAPA B, NAMPA B and NAPAH B, computed at the RI-CC2/cc-pVDZand MS-CASPT2/SA-CASSCF(14,12)/cc-pVDZ levels. The ground state minimum structures were optimized at the RI-CC2/cc-pVDZ level of theory. (*Adiabatic*) and [**nuclear zero point energy corrected adiabatic**] transitions are given. The RI-CC2/cc-pVDZ method was used for the geometrical optimization of the first excited state structures, as well as for the calculation of the Hessian matrix in the ground and first excited state. All values are in eV.

		RI-CC2	CASPT2	Exp.
NAPA B	$\pi \pi^{*}$	5.176 (*5.002*) [**4.797**]	4.761 (*4.606*)	4.663 [17]
	$n\pi_{\left(II\right)}^{*}$	5.606	5.530	
	$n\pi_{\left(I\right)}^{*}$	5.771	5.620	
	$\pi \pi^{*}$	6.460	7.764	
NAPMA B	$\pi \pi^{*}$	5.176 (*5.000*) [**4.941**]	4.759 (*4.610*)	4.662 [18]
	$n\pi_{\left(II\right)}^{*}$	5.695	5.597	
	$n\pi_{\left(I\right)}^{*}$	5.785	5.620	
	$\pi \pi^{*}$	6.447	7.751	
NAPAH B	$\pi \pi^{*}$	5.178 (*5.003*) [**4.833**]	4.760 (*4.609*)	4.661 [25]
	$n\pi_{\left(I\right)}^{*}$	5.769	5.615	
	$n\pi_{\left(II\right)}^{*}$	5.813	5.774	
	$\pi \pi^{*}$	6.484	7.796	

**Table 3:** Relative energies (ΔE) of $M_{\pi \pi^{*}}$ and M_(*II*)_ minima in NAPA B and NAPMA B. The three torsional angles, $\vartheta_{C}$, $\vartheta_{N}$ and $\omega_{2}$, shown in Figure 3 describe the deplanarization of the second amide group. Energies are in eV, angles in degrees.

	NAPA B M_(*II*)_				NAPMA B M_(*II*)_			
	ΔE	$\vartheta_{C}$	$\vartheta_{N}$	$\omega_{2}$	ΔE	$\vartheta_{C}$	$\vartheta_{N}$	$\omega_{2}$
$M_{\pi \pi^{*}}$	0.00	−178.7	−159.4	−6.9	0.00	−178.3	−178.5	1.6
*a*1	−0.71	−127.6	−122.3	33.2	−0.72	−121.8	−147.8	61.7
*a*2	−0.69	−131.5	−124.9	−1.6	−0.63	−130.7	−139.5	−7.1
*a*3	−0.64	−128.2	130.7	56.9	−0.62	−128.4	140.2	50.0
*b*1	−0.73	125.4	120.7	−36.8	−0.74	121.4	143.4	−61.7
*b*2	−0.72	129.9	125.7	3.5	−0.68	128.2	140.9	9.2
*b*3	−0.69	125.6	−127.4	−44.9	−0.67	125.7	−139.2	−41.5

**Table 4:** Energies (in eV) of a set of $\pi \pi^{*}$/$n\pi_{\left(II\right)}^{*}$ conical intersection (CI) geometries of NAPA B and NAPMA B relative to corresponding $M_{\pi \pi^{*}}$ minimum. Included are minimum energy CI (MECI), minimum distance CI (MDCI) and CI points obtained by linear interpolation (LIP) between $M_{\pi \pi^{*}}$ and six $M_{n\pi_{\left(II\right)}^{*}}$ minima. Relative energies calculated assuming a harmonic approximation for the $\pi \pi^{*}$ surface (HAR) and RMSD (Å) between CI and $M_{\pi \pi^{*}}$ structures. Classically-accessible geometries are given in bold.

	NAPA B CI_(*II*)_			NAPMA B CI_(*II*)_		
	LIP	HAR	RMSD	LIP	HAR	RMSD
MECI*a*	**0.13**	**0.13**	**0.09**	0.17	0.34	0.20
*a*1	**0.21**	**0.20**	**0.09**	0.32	0.38	0.17
*a*2	**0.19**	**0.17**	**0.06**	**0.24**	**0.25**	**0.12**
*a*3	**0.25**	**0.26**	**0.07**	**0.25**	**0.26**	**0.09**
MECI*b*	0.16	0.95	0.19	0.17	0.66	0.25
*b*1	0.42	0.78	0.17	0.45	0.88	0.18
*b*2	0.35	0.34	0.15	0.33	0.33	0.24
*b*3	0.29	0.24	0.15	0.32	0.43	0.21
MDCI	0.22	0.25	0.01	0.28	0.31	0.01

**Table 5:** Relative energies (eV) and RMSD (Å) of a set of $\pi \pi^{*}$/$n\pi_{\left(II\right)}^{*}$ MECI geometries in NAPAH B with respect to the $M_{\pi \pi^{*}}$ geometry.

	LIP	RMSD
MECI*a*1	0.22	0.18
MECI*a*2	0.23	0.14
MECI*a*3	0.25	0.24
MECI*b*	0.24	0.20
